# “Pass, fail” – On Standard Setting Procedures for the Assessment of Practical Skills at Medical Schools in Germany, Austria, and Switzerland

**DOI:** 10.3205/zma001049

**Published:** 2016-08-15

**Authors:** Daniel Bauer, Sören Huwendiek, Maren März

**Affiliations:** 1 Klinikum der Universität München, Institut für Didaktik und Ausbildungsforschung in der Medizin, München, Deutschland; 2 Universität Bern, Institut für Medizinische Lehre, Bern, Schweiz; 3 Gesellschaft für Medizinische Ausbildung e.V., Ausschuss Prüfungen, Erlangen, Deutschland; 4 Charité Universitätsmedizin Berlin, Referat für Studienangelegenheiten, Berlin, Deutschland

## Short report

The members of the GMA committee on assessment have the common goal to make exams in the training and continuing education of health professionals in Germany, Austria and Switzerland (D/A/CH countries) as appropriate as possible. For this purpose, they engage in a discourse with each other, considering both scientific research and regional/local contexts. The committee is pleased to present in the present special issue its views on practical skills from the assessment perspective.

The teaching of practical skills is now well-established in the medical programs in the D/A/CH region, reflected in the national catalogues of learning objectives: The Swiss Catalogue of Learning Objectives for Undergraduate Medical Training (SCLO) [1], the Austrian Competence Catalogue for Medical Skills (ÖKÄF) [2], and the German National Competence Based Catalogues of Learning Objectives for Undergraduate Medical Education (NKLM) [3].

The assessment of practical skills, too, is largely established. As early as 2007, over 70% of Germany’s medical programs featured practical skills assessment, most of which in OSCE format [4], and since 2011, an OSCE complements the Swiss Federal Licensing Examination and must be passed independently of the MCQ examination [5]. Besides OSCE, the oral-practical examination [6] is a common format, but also OSPEs (Objective Structured Practical/Preclinical Exams) are used [7]. This shows that practical examinations in medical education are indeed varied and widespread, but what about their quality?

Yudkowsky lists various criteria compromising construct validity – i.e., the meaningful interpretability of the data obtained in an exam [8]. As is the case with exams of other formats, it is necessary in the assessment of practical skills such as the OSCE to prevent construct underrepresentation by providing sufficient and representative examination content and to minimize construct-irrelevant variance, i.e., to ignore, in interpreting exam performance, such information not relating to the subject matter under scrutiny. Quality-maintaining measures are being taken in many programs, such as blueprinting (selection of adequate, representative exam content), review (e.g., with respect to wording of tasks and checklists, and to anticipate task difficulty), as well as training of examiners and possibly, simulated patients involved.

However, it seems that one aspect of quality assurance has been receiving only little attention: the deliberate setting of grades and pass/fail boundaries. In a commentary recently published, Tekian & Norcini advocate questioning the traditional, ubiquitous 60% pass boundary, and recommend to apply methods based on the assessment content in order to determine pass/fail and grades, for only this way, comparability can be established between test cohorts [9]. The challenge is, therefore, to operationalize these general requirements within the context of one’s actual practical skills assessment (the so-called standard setting) [10], [11].

So far, there is little data on the use of these criterion-oriented standard setting procedures in practical examinations at the medical schools of the D/A/CH countries. Accordingly, we exemplarily analysed 20 study and/or exam regulations or equivalent documents available on the internet relating to medical curricula in Germany, including all model programs. It turned out that the majority of programs apply the 60% pass boundary to practical exams [12], [13], [14], while some do not specifically address grades and pass criteria for practical exams [15], [16], [17], [18], [19] beyond perhaps referring to general directives (e.g., §13 (2) ÄAppO).

Nevertheless, this common 60% rule does not rule out criterion-oriented standard setting approaches. Tekian & Norcini explicitly mention procedures under which a criterion-oriented pass/fail boundary can be rescaled to 60%, so that - if necessary - the traditional 60% can be retained. In fact, the Hannover medical program uses such a procedure in which an examination board determines, according to the [anticipated] difficulty and scope of the tasks, the maximum score from which at least 60% must be achieved to pass an exam [20]. Some regulations delegate the burden on the examiners [21], [22], [23], [24], occasionally with the caution to apply "proper methods" [25], or have an examination committee in place [26], [27], [28] to define pass and grade criteria. One faculty explicitly allows criterion-oriented methods for determining the pass boundaries [29], while others require only the disclosure of pass criteria at the beginning of the respective term, without the obligation to further specify them [30], [31].

The Medical Universities of Vienna [32] and Graz [33] use norm-oriented procedures while Innsbruck allows some adaptation depending on task difficulty [34]. The pass boundary for the clinical skills part of the Swiss Federal Licensing Examination [35] is determined with the borderline regression method, which is also true, e.g., for the OSCE in Basel’s medical program [36]. 

In summary, it is apparent from the examined documents that the regulations on pass and grade determination are heterogeneous. In some locations, detailed rules have been formulated, but many regulations handle pass/fail and grades rather superficially, which of course does not mean that decisions are not made very consciously in the local reality. Interpreting the documents was not always easy, and there may be passages that have been misinterpreted. Likewise, due to the selection of the studied faculties, interesting approaches may have been overlooked. For instance, dental and veterinary regulations were not analysed for this work. Still, our findings coincide well with those of Härtl et al., who conducted a survey on the assessment of communication skills at German-speaking medical schools. From 31 OSCEs with communication as test objective, 21 pegged the pass boundary to a fixed score or percentage, 5 applied the borderline regression method, 2 used Angoff’s method, and in 6 OSCEs, the method used was unknown (some multiple responses) [37].

On behalf of the committee we recommend to question the local regulations used to determine pass/fail and scoring criteria for practical exams, to seek the discourse, and to share and discuss experiences with the scientific community. Procedures used to define pass criteria and scoring should be based on examination content. This would add to the validity of pass/fail decisions of practical exams and better ensure fairness to examinees. The members of the GMA committee on assessment are gladly available for this discourse as discussion partners.

## Competing interests

The authors declare that they have no competing interests.
